# Neuropilin Regulation of Angiogenesis, Arteriogenesis, and Vascular Permeability

**DOI:** 10.1111/micc.12124

**Published:** 2014-05-22

**Authors:** Alice Plein, Alessandro Fantin, Christiana Ruhrberg

**Affiliations:** UCL Institute of Ophthalmology, University College LondonLondon, UK

**Keywords:** angiogenesis, VEGF, VEGF-A, SEMA3A, neuropilin, NRP1, VEGFR2, endothelial cells, angiogenesis, arteriogenesis, vascular permeability

## Abstract

The formation of the cardiovasculature, consisting of both the heart and blood vessels, is a critical step in embryonic development and relies on three processes termed vasculogenesis, angiogenesis, and vascular remodeling. The transmembrane protein NRP1 is an essential modulator of embryonic angiogenesis with additional roles in vessel remodeling and arteriogenesis. NRP1 also enhances arteriogenesis in adults to alleviate pathological tissue ischemia. However, in certain circumstances, vascular NRP1 signaling can be detrimental, as it may promote cancer by enhancing tumor angiogenesis or contribute to tissue edema by increasing vascular permeability. Understanding the mechanisms of NRP1 signaling is, therefore, of profound importance for the design of therapies aiming to control vascular functions. Previous work has shown that vascular NRP1 can variably serve as a receptor for two secreted glycoproteins, the VEGF-A and SEMA3A, but it also has a poorly understood role as an adhesion receptor. Here, we review current knowledge of NRP1 function during blood vessel growth and homeostasis, with special emphasis on the vascular roles of its multiple ligands and signaling partners.

## INTRODUCTION

The cardiovascular system forms during embryonic development to supply growing organs with oxygen and nutrients via a hierarchical blood vessel network, with blood flow generated by the pumping heart. Developmental blood vessel growth occurs through the complementary processes of vasculogenesis to form new vessels from single cell precursors and angiogenesis to expand existing vessels by sprouting growth or intussusception (reviewed in refs [16,64,65]). These processes are accompanied by arteriovenous specification to increase luminal caliber and allow the separation of blood leaving the heart from blood that is returned to it. In addition, endothelial cells acquire organ-specific properties. Thus, endothelial cells in the glomerulus of the kidney become fenestrated to allow passage of solutes, whereas endothelial cells of the brain and retina establish tight junctions to form blood barriers and maintain organ homeostasis (reviewed in refs [64,67]). In most organs, however, intermediate barrier properties are established during embryonic development to enable appropriate serum efflux and extravasation of circulating immune cells during postnatal life.

After birth, angiogenesis continues to support growing tissues, but most blood vessels become quiescent during adulthood. The reactivation of quiescent vessels occurs only under specific circumstances in adults, for example in the cycling uterus and ovary, in the placenta during pregnancy, and in skeletal muscle to support exercise-induced muscle growth (reviewed in refs [16,41,42]). Angiogenesis is also stimulated after injury to promote tissue repair through an increased vascular supply, but this response can be detrimental, for example, in ocular pathologies such as proliferative diabetic retinopathy or the “wet” form of age-related macular degeneration, in which tissue ischemia leads to the formation of ectopic and leaky blood vessels (reviewed in ref. [16]). Moreover, tumor angiogenesis can promote tumor growth, thus contributing to cancer progression. In such diseases, neo-angiogenesis typically leads to the formation of abnormal vessels with increased vascular permeability. Although vascular permeability is beneficial after acute tissue injury through the delivery of coagulation factors, antibodies, and cytokines, chronic permeability can cause pathological tissue edema.

In this review, we will describe current knowledge of vascular signaling pathways mediated by the transmembrane protein NRP1, a bifunctional receptor for the secreted glycoproteins VEGF-A and SEMA3A that can additionally promote cell-matrix interactions and adhesion (reviewed in [26,71]). We will further discuss the relevance of these NRP1 pathways for the design of novel therapies aimed at normalizing pathological vasculature and stimulating the functional revascularization of ischemic organs.

## DOMAIN STRUCTURE OF NRP1 AND ITS HOMOLOG NRP2

**Figure 1:**
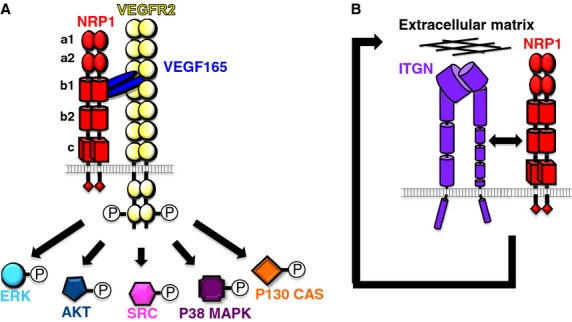
NRP1-regulated signaling pathways in endothelial cells *in vitro*. (A) VEGF165 induces complex formation between NRP1 and VEGFR2 to enhance VEGFR2 signaling in endothelial cells *in vitro*. In particular, biochemical studies suggested possible roles for NRP1 in the VEGF-mediated induction of pathways involving the activation of ERK, AKT, SRC, P38 MAPK, and p130 CAS. (B) NRP1 regulates integrin-dependent fibronectin remodeling in primary arterial endothelial cells and tumor cells in a mechanism that depends on the NRP1 cytoplasmic domain, but is thought to be VEGFR2 independent.

NRP1 is a single-pass transmembrane glycoprotein of 130 kDa [26] that was originally discovered in the developing frog nervous system as an axonal adhesion protein [82] and subsequently in mammals as a receptor for secreted axon guidance cues of the class 3 semaphorin family such as SEMA3A [38,49]. In addition to binding semaphorins, NRP1 also binds VEGF165, an isoform of the vascular endothelial growth factor VEGF-A that arises through alternative splicing [32,75]. These multiple NRP1 interactions are facilitated by a large extracellular domain of 860 amino acids that is organized into five domains, termed a1, a2, b1, b2, and c (Figure1A) [26,71]. Whereas the a and b domains bind ligands, the c domain promotes oligomerization.

The class 3 semaphorins bind the a1 and a2 domains of NRP1, and the disruption of this interaction leads to axonal patterning defects without affecting vascular development [36,87]. The b1 and b2 domains mediate binding to the polysaccharide heparin sulfate and VEGF165, and they additionally promote cell adhesion [35,55]; however, the VEGF165 binding sites are distinct from the cell adhesion sites [53,73]. More recently, structural studies of the b1 domain have identified key residues important for ligand interactions in a VEGF_165_-binding pocket, such as tyrosine (Y) 297 and aspartic acid (D) 320 [43,86,88]. In addition to its extracellular domains, NRP1 has a short, 39 amino acid-long intracellular/cytoplasmic tail that is thought to lack catalytic activity (Figure1A). However, the C-terminal SEA motif in NRP1's intracellular domain can recruit synectin, a modulator of endocytic trafficking that is also known as GIPC1 or NIP [4,8,17,28,51,69].

The neuropilin family also includes NRP2, which has roughly 44% amino acid homology with NRP1 and an identical domain structure [14]. NRP2 also binds VEGF-A isoforms and class 3 semaphorins, with preferential binding of VEGF145 and SEMA3F over VEGF165 and SEMA3A, respectively [33]. Although semaphorin signaling through NRP2 plays an important role in neurons, NRP2 is dispensable for blood vascular patterning in mice. NRP2 knockout mice do, however, have mild lymphatic defects [97]. Thus, in contrast to NRP2, NRP1 has pleiotropic and essential roles in the blood vasculature and is, therefore, the focus of this review.

## NRP1 IN VASCULAR DEVELOPMENT

During development, NRP1 expression is prominent in growing blood vessels, for example on the endothelial cells of capillaries, arteries, and veins in the postnatal mouse retina and in capillaries in the mouse embryo hindbrain on embryonic day (E) 11.5 [21–23]. The importance of NRP1 for vascular growth was first demonstrated by NRP1 overexpression studies in the mouse embryo, which led to the excessive growth of vessels that were leaky and hemorrhagic [48]. In contrast, loss of NRP1 function in mouse embryos reduces vessel sprouting in organs vascularized by angiogenesis, especially the brain and spinal cord, and it also impairs the remodeling of the heart outflow tract, leading to embryonic lethality by E12.5 in the CD1 mouse background [24,31,47]. In the C57Bl/6 background, NRP1 loss additionally impairs yolk sac vascularization and, therefore, causes death at E10.5 [44], while the JF1 background is compatible with survival up to E14.5 [9,70]. These observations suggest the existence of important, but as yet unidentified genetic modifiers for NRP1 signaling.

In zebrafish, two NRP1 homologs have been identified: *nrp1a*, whose sequence is phylogenetically more similar to the mammalian gene, and *nrp1b*, which originates from an early duplication of the NRP1 gene during the evolution of teleost fish [6,57,96]. Knockdown studies with morpholinos for either gene reported impaired intersegmental vessel patterning and resulted in improper arteriovenous connections, suggesting that both *nrp1* genes are required for vascular development in fish [52,57,90].

Several studies had raised the possibility that non-endothelial NRP1 regulates angiogenesis. For example, NRP1 is expressed on multiple cell types in an angiogenic setting. Thus, NRP1 is expressed by the tumor cells as well as the tumor vasculature (e.g., [76)], and in the endothelium alongside neural progenitors and tissue macrophages during hindbrain angiogenesis [24]. Moreover, the angiogenic brain defects of endothelial NRP1 knockouts are milder than those of full knockouts [24]. Using *Cre-LoxP* technology to create cell type-specific NRP1 mutants in the hindbrain angiogenesis model, we recently examined the dependence of vascular development on endothelial versus nonendothelial NRP1. Surprisingly, we found that, NRP1 was required only on the vascular endothelium for normal angiogenesis, despite its abundant expression by nonendothelial cells [24]. Genetic mosaic experiments further demonstrated that NRP1 is required within the angiogenic endothelium to generate the specialized tip cells that lead vessel sprouts [24].

## NRP1 LIGANDS IN ANGIOGENESIS: VEGF165

Based on prior biochemical and tissue culture studies, vascular NRP1 functions are widely thought to be mediated by its binding to VEGF165, as this is the VEGF-A isoform with the strongest affinity for NRP1. However, our genetic mouse studies had raised the possibility that NRP1 can also function in angiogenic endothelial cells in a VEGF-independent manner. Most notably, mice expressing only VEGF120 at the expense of NRP1-binding VEGF (*Vegfa*^*120/120*^ mice) display vascular defects that are different to those of full NRP1 knockout mice; for example, the perisomitic vessels branch normally in E10.5 *Nrp1*-null mutants, whereas their branching is reduced in *Vegfa*^*120/120*^ mutants [68]. Furthermore, the vascular defects in the E12.5 hindbrain differ qualitatively in both types of mutants: Although *Nrp1*-null brains show a severe reduction in vessel branching specifically in the subventricular zone, with vessels terminating in bulbous vascular tufts [24,31], *Vegfa*^*120/120*^ brains show a milder reduction in vessel branching that is accompanied by increased vascular diameter and impaired sprouting both into and within the brain [23,68]. In fact, the vascular defects of *Vegfa*^*120/120*^ mutants are better explained by the differential affinity of VEGF-A isoforms for the extracellular matrix than their distinct receptor binding properties. Thus, VEGF121 is highly and VEGF165 partly diffusible, whereas VEGF189 is retained in the matrix, unless released by proteases [61]. These binding properties are thought to allow the isoforms to form chemotactic gradients critical for normal vascular morphogenesis [30,68].

To directly evaluate the importance of VEGF binding to NRP1 for angiogenesis *in vivo*, we recently examined *Nrp1*^*Y297A/Y297A*^ mice expressing NRP1 with a point mutation in the VEGF binding pocket [20], as the mutated residue was previously shown to be important for high affinity VEGF binding by NRP1 [40,43]. The *Nrp1*^*Y297A/Y297A*^ mutants do not show the severe embryonic vascular defects of full or endothelial-specific NRP1 knockouts [20]. Hence, VEGF binding to NRP1 is not essential for embryonic angiogenesis, and, therefore, NRP1 must have VEGF-independent roles in angiogenesis that likely synergize with its known role as a VEGFR2 coreceptor.

Although VEGF binding to NRP1 appeared to be largely dispensable for embryonic angiogenesis, the study of newborn and adult *Nrp1*^*Y297A/Y297A*^ mice revealed essential roles for NRP1 in postnatal angiogenesis and arteriogenesis in the heart and retina, and in pathological neovascularization in a model of neonatal eye disease [20]. In these settings, the vascular phenotypes of *Nrp1*^*Y297A/Y297A*^ mutants closely resemble the ones of *Vegfa*^*120/120*^ mice lacking NRP1-binding VEGF, suggesting that NRP1-mediated VEGF-signaling is more important for perinatal and pathological neo-vascularization than embryonic vascular development. However, because the targeting strategy used to create the *Nrp1*^*Y297A/Y297A*^ mice inadvertently also reduced NRP1 expression, further studies will be required to unequivocally define the specific contribution of VEGF165 signaling through NRP1 to both embryonic and postnatal angiogenesis, as well as pathological neovascularization.

## NRP1 LIGANDS IN ANGIOGENESIS: SEMA3A

As mentioned above, the extracellular NRP1 domain has distinct VEGF165 and semaphorin binding domains to which these two ligands bind noncompetitively [3]. However, *in vivo* studies showed that neither SEMA3A nor semaphorin signaling through NRP1 and NRP2 are essential for embryonic angiogenesis in the mouse [36,87]. Moreover, abolishing both SEMA3A and VEGF164 in mice impairs brain vascularization no differently than loss of VEGF164 alone [87]. These observations argue against an obvious role for semaphorin signaling through NRP1 in the vasculature of developing mice. In zebrafish, however, decreasing the levels of either of two SEMA3A orthologs impairs vascular development [74,84]. The exogenous delivery of SEMA3A also disrupts VEGF-A-mediated angiogenesis in the chick chorioallantoic membrane assay [1]. It is not yet known why SEMA3A affects angiogenesis in developing fish and chick, but is dispensable for angiogenesis in mouse embryos. In addition, SEMA3A has been reported to also affect pathological angiogenesis in mice. For example, SEMA3A prevents vascular regeneration in a mouse model of oxygen-induced retinopathy [45] and inhibits tumor angiogenesis by eliciting endothelial cell apoptosis and normalizing the pericyte coverage of tumor vessels [12,54]. SEMA3A-induced tumor vessel normalization might be indirectly caused by SEMA3A-mediated recruitment of a subset of NRP1-expressing monocytes that secrete several factors involved in vessel maturation [11,98].

## NRP1 LIGANDS IN ANGIOGENESIS: INTEGRIN LIGANDS IN THE EXTRACELLULAR MATRIX

In agreement with the original discovery of NRP1 as an adhesion molecule in the nervous system [82], NRP1 was shown to promote endothelial cell attachment to extracellular matrix [59] and to directly interact with integrins *in vitro* (Figure1B) [27,85]. Moreover, *α*v*β*3 integrin can negatively regulate VEGF-A-mediated angiogenesis by limiting the interaction of NRP1 with VEGFR2 [66]. The domains that enable NRP1 to modulate adhesion to heterologous, but unidentified proteins on neighboring cells reside in the b1 and b2 domains [73]. It is not yet known whether NRP1-mediated intercellular or cell-matrix adhesion regulates the interaction of blood vessels with their environment during angiogenesis *in vivo*. Nevertheless, NRP1-mediated extracellular matrix remodeling takes place in primary arterial endothelial cells and tumor cells and appears to be mediated by the NRP1 cytoplasmic tail without requirement for VEGFR2 activation by VEGF [85,95] (Figure1B).

## MECHANISM OF NRP1 SIGNAL TRANSDUCTION IN THE VASCULATURE: ASSOCIATION WITH VEGFR2

The cytoplasmic tail of NRP1 lacks any known catalytic activity, suggesting that NRP1 transduces signals in the vasculature through a coreceptor. This would be analogous to how NRP1 conveys semaphorin signals in the nervous system, where A-type plexins function as a NRP1-coreceptor and signal transducer [60]. Candidates for NRP1 signal transduction in endothelial cells are the VEGF-A receptor tyrosine kinases VEGFR1 (FLT1) and VEGFR2 (KDR), as both have been shown to interact with NRP1 *in vitro*, and because NRP1 increases VEGFR2 phosphorylation [25,34,77,93]. In fact, the most widely accepted model of NRP1 function in angiogenesis postulates that it forms a VEGF165-dependent complex with VEGFR2 to enhance the activation of a wide variety of intracellular signal transduction pathways, including those that involve (ERK) 1 and 2 (MAPK1 and MAPK3), the serine/threonine protein kinase AKT1, the avian sarcoma viral oncogene homolog SRC, p38 MAPK (MAPK14) and the P130 CRK-associated substrate (CAS), also known as breast cancer anti-estrogen resistance 1 (BCAR1) (Figure1A) [1,5,19,46,51,89].

The VEGF165-mediated interaction between NRP1 and VEGFR2 may occur in *cis*, if both receptors are coexpressed on the same endothelial cell, or in *trans*, if VEGFR2 is expressed by an endothelial cell and NRP1 by an adjacent endothelial or nonendothelial cell. Thus, it has been proposed that endothelial VEGFR2 may interact with tumor NRP1 in *trans* [76]. However, the significance of *trans* interactions has not yet been established for tumor angiogenesis, nor is it known if *trans* interactions take place between endothelial and mural cells in the dorsal aorta [62,94]. We have recently shown that a *trans* interaction between nonendothelial NRP1 and vascular endothelium is dispensable for angiogenesis in the developing brain, where endothelial NRP1 alone is essential for normal vessel growth [24,36]. Thus, the ablation of NRP1 from the neural progenitors that secrete VEGF-A for vessel sprouting or from the macrophages that promote vascular anastomosis did not compromise brain angiogenesis [24] (see above).

Recent *in vitro* studies suggest that NRP1-VEGFR2 complex formation requires the C-terminal SEA motif in the NRP1 cytoplasmic tail and its intracellular binding partner synectin [63]. This complex is thought to promote ligand-dependent endocytosis of VEGFR2-NRP1 complexes in cultured endothelial cells [69]. Overexpression studies with VEGF receptors and fluorophore-linked RAB proteins in porcine aortic endothelial cells, which lack endogenous VEGF receptor expression, further suggested that the synectin-binding motif of NRP1 promotes sequential, NRP1-mediated VEGFR2 recycling through specific subsets of RAB-vesicles after VEGF165 stimulation [4]. The latter study concluded that NRP1-mediated VEGFR2 recycling controls signaling output from VEGF-A-activated VEGFR2 complexes [4].

Although NRP1-mediated VEGFR2 trafficking was initially hypothesized to promote angiogenesis (e.g., ref [4]), the analysis of mice lacking the cytoplasmic tail of NRP1 showed that this domain is dispensable for developmental angio-genesis in the mouse [22], which involves blood vessel sprouting, migration, and vascular anastomosis. In contrast, the NRP1 cytoplasmic tail is essential for efficient arteriogenesis, a process that involves luminal vessel growth [51]. Thus, loss of the NRP1 cytoplasmic tail impairs arterial morphogenesis during development and in adult hindlimb ischemia, similar to the loss of synectin [15], which serves as an adaptor to link VEGFR2 to myosin VI motors for endocytic trafficking into EEA1+ endosomes [50]. Mechanistically, these studies suggest a model in which the cytoplasmic tail of NRP1 mediates VEGF165-stimulated VEGFR2 cotrafficking with NRP1 via a synectin/myosin VI complex into EEA1+ endosomes, where VEGFR2 is protected from dephosphorylation by the phosphatase PTP1b to maximize the phosphorylation of ERK1/2 [51], a previously identified target of NRP1-dependent VEGFR2 signal transduction (Figure1A) [5].

## VEGFR2-INDEPENDENT NRP1 SIGNALING IN ENDOTHELIAL CELLS

Several tissue culture studies raised the possibility that NRP1 can promote endothelial migration and extracellular matrix remodeling independently of VEGFR2. First, fusion of the extracellular domain of the EGF receptor to the transmembrane and cytoplasmic domains of NRP1 creates a chimeric receptor that promotes endothelial cell migration in response to EGF, independently of VEGFR2 [91]. Second, NRP1 is required for the VEGF-induced migration of primary venous endothelial cells via activation of p130CAS, and overexpression studies raise the possibility that this may occur independently of VEGFR2 phosphorylation [19]. Third, NRP1 promotes adhesion of endothelial cells to low concentrations of extracellular matrix component and integrin ligand fibronectin, independently of VEGFR2 [59] in a mechanism that involves the NRP1 cytoplasmic tail [85]. Because the cytoplasmic domain of NRP1 is dispensable for developmental angiogenesis [22], these tissue culture studies may have identified signaling pathways that are selectively important for endothelial cell migration in specific circumstances, for example in pathological situations (although *in vivo* evidence for this suggestion is still lacking). In contrast to mice, zebrafish show defective vascular development in the absence of the SEA motif in the NRP1 cytoplasmic domain [90] or its interactor synectin [15]. These species differences are reminiscent of the selective importance of SEMA3A for zebrafish, but not mouse intersomitic vessel development (see above). In summary, it is clear that NRP1 plays an essential role in angiogenesis, but conflicting data obtained in tissue culture, zebrafish, and mouse models need to be resolved to fully understand the signaling mechanism of NRP1 during angiogenesis and other vascular functions *in vivo*.

## ROLE FOR NRP1 IN ARTERIOVENOUS PATTERNING

NRP2 is enriched in the venous and NRP1 in the arterial parts of vascular networks in chick, mouse and zebrafish [22,39,44,57]. Although *Nrp2*-null mice develop to term without obvious arteriovenous or other type of blood vascular defects [97], specific arterial markers are missing from arterioles and arteries in full and endothelial-specific *Nrp1* knockouts [44,58]. The arterial differentiation defects observed in limb skin are thought to be due to defective VEGF-A signaling [58]. In agreement, both the loss of NRP1 binding VEGF-A isoforms and, inversely, the loss of VEGF-A binding to NRP1 impair arterial development in the retina and the heart in a similar manner [10,80]. Endothelial NRP1 deficiency also precludes normal remodeling of the arterial pole of the developing heart from a common outflow tract into the pulmonary artery and aorta [36]. This observation has been proposed to be due to defective VEGF-A binding to NRP1 on outflow tract endothelium [36], agreeing with the observation that *Vegfa*^*120/120*^ mice lacking NRP1-binding VEGF165 have similar defects [79]. However, loss of VEGF-A binding to NRP1 does not affect embryonic viability, which depends on a properly remodeled outflow tract [20], and mice lacking semaphorin signaling through NRP1 also have defective outflow tract remodeling [36]. Accordingly, further work is needed to define the specific roles of NRP1 in endothelial cells and other cell types that interact with the endothelium during outflow tract remodeling, such as neural crest cells.

Despite its essential role in arterial morphogenesis, the NRP1 cytoplasmic domain is dispensable for NRP1-mediated arterial differentiation in the retina; instead, the NRP1 cytoplasmic domain promotes the spatial segregation of arteries and veins to regulate arteriovenous patterning in this tissue. Specifically, retinal arteries and veins cross over each other in the *Nrp1*^*cytoΔ/Δ*^ mutants at an abnormally high frequency [22]. In humans, similar arteriovenous crossings, in the presence of additional systemic factors, are thought to increase the likelihood of developing branch retinal vein occlusion, in which compression of the vein by the artery disrupts retinal blood flow [7,92]. Arteriovenous crossings have also been identified in the retina of mice with haploinsufficient expression of VEGF-A in neural progenitors [37], suggesting that NRP1 conveys VEGF-A signals through its cytoplasmic domain to implement the spatial separation of arteries and veins in the retina. However, is not yet known if this process requires NRP1 expression in endothelial or mural cells, as the latter are also known to express NRP1 in certain situations [62,94].

## NRP1 IN VASCULAR PERMEABILITY

Although VEGF-A is best known as an angiogenic growth factor, it was first described as a vascular permeability factor, because it disrupts endothelial barrier function in tumors and increases the leakage of serum proteins and therefore interstitial pressure [72]. In an inflammatory setting, the effect of VEGF-A on permeability is beneficial, because it enables the delivery of clotting factors and antibodies, but in chronic conditions such as age-related macular degeneration it is detrimental, because tissue swelling impairs vision. Mechanistically, VEGF-A-mediated VEGFR2 activation induces the internalization of VE-cadherin from adherens junctions [18] and increases paracellular permeability in a SRC- and T-cell-specific adapter (TSAD) (SH2D2A)-dependent mechanism [29,81]. Supporting an additional role of NRP1 in VEGF-mediated vascular permeability, mice that overexpress soluble NRP1 in the skin show reduced leakage in response to VEGF165 in the Miles assay, a technique that measures vascular leak in the skin [56]. Moreover, antibody neutralization of NRP1 attenuates VEGF165-induced vascular leakage in lung tissue [5]. Interestingly, C-end rule peptides with a C-terminal arginine residue in a configuration similar to that of VEGF-A bind NRP1 to induce vascular permeability [83], and this property may be exploited to enhance tumor penetration of chemotherapeutic drugs [2].

It is not yet clear how NRP1 and VEGFR2 cooperate to regulate vascular permeability. On the one hand, NRP1 and VEGFR2 need to be coexpressed in porcine aortic endothelial cells to restore the VEGF165-induced decrease in transendothelial electrical resistance, an inverse indicator of permeability [5]. On the other hand, some experiments suggest that NRP1 promotes permeability independently of VEGFR2. First, blocking VEGFR2 in pulmonary artery endothelial cells does not prevent the VEGF165-mediated decrease in transendothelial electrical resistance, while blocking NRP1 does [5]. Second, a mutant form of VEGF165 lacking the ability to bind VEGFR2, but predicted to still bind NRP1, can also induce vascular permeability [78].

In addition to binding VEGF165, NRP1 can also bind SEMA3A to induce permeability in the Miles assay that measures vascular permeability in the skin [1]. In agreement, a recent study revealed that SEMA3A is induced in the neural retina of diabetic humans and mice, where it promotes vascular leakage in a NRP1-dependent way [13]. SEMA3A binding to NRP1 may, therefore, provide a therapeutic target in diabetic retinopathy. It has not yet been investigated if this pathway is also involved in other diseases in which elevated vascular permeability is a contributing factor, such as cancer, stroke, and other eye diseases. Moreover, further studies are required to better discern the precise downstream mechanisms that enable the VEGF-A isoforms and semaphorins to regulate vascular permeability *in vivo*.

## PERSPECTIVE

Due to its pleiotropic roles in angiogenesis, arteriogenesis, and vascular permeability, NRP1 provides a promising molecular target for diseases involving aberrant vascular growth or excessive vascular leak. In particular, a better understanding of NRP1 signaling may enable the design of therapies that can revascularize ischemic tissues via VEGF-A-stimulated angiogenesis and arteriogenesis, but avoid induction of excess vascular leak.

## Abbreviations

AKT1: RAC-alpha serine/threonine-protein kinase
BCAR1: breast cancer anti-estrogen resistance 1
EEA1: early endosomal antigen 1
EGF: epidermal growth factor
ERK: extracellular signal-regulated kinase
MAPK: mitogen-activated protein kinase
NRP: neuropilin
p130CAS: CRK associated substrate
RAB: Ras-related proteins in brain
SEA: serine-glutamic acid-alanine
SEMA3A: semaphorin
SRC: proto-oncogene tyrosine-protein kinase Src
TSAd: T-cell-specific adapter
VEGF-A: vascular endothelial growth factor
VEGFR: vascular endothelial growth factor receptor
